# The Online Life Story Book: A randomized controlled trial on the effects of a digital reminiscence intervention for people with (very) mild dementia and their informal caregivers

**DOI:** 10.1371/journal.pone.0256251

**Published:** 2021-09-15

**Authors:** Teuntje R. Elfrink, Christina Ullrich, Miriam Kunz, Sytse U. Zuidema, Gerben J. Westerhof

**Affiliations:** 1 Department of Psychology, Health and Technology, University of Twente, Enschede, The Netherlands; 2 Department of General Practice and Elderly Care Medicine, University of Groningen, University Medical Center Groningen, Groningen, The Netherlands; Public Library of Science, UNITED STATES

## Abstract

This paper describes a randomized controlled trial on the Online Life Story Book (OLSB), a digital reminiscence intervention for people with (very) mild dementia living at home. The aim of the study was to investigate the effectiveness of the OLSB on (i) neuropsychiatric symptoms (NPS) in persons with dementia and (ii) the distress and quality of life (QOL) of primary informal caregivers. A randomized controlled trial with individual randomization to one of two conditions was conducted: 1) intervention “Online Life Story Book”; 2) wait list control condition. In the intervention OLSB, a trained volunteer guided the participants through the process of creating an OLSB in approximately 5 meetings within a period of 8–10 weeks. Participants in the control condition received care as usual while they waited for 6 months before starting. Outcomes on NPS and distress and QOL of the informal caregiver were assessed at baseline (baseline, T0), 3 months (T1) and 6 months (T2) post baseline. Of the 42 persons with dementia, 23 were female and 19 were male. They had a mean age of 80 years, ranging from 49 to 95. The total drop-out rate was 14.3 percent. Small but insignificant effects on NPS, caregiver distress and QOL of caregivers were found with the exception of self-rated caregiver distress that reduced significantly during the intervention. One reason to explain the results might be that the included participants were in relatively good health. Practical challenges during the intervention could have affected the results as well. It might also be that the intervention caused effects on other outcomes than NPS and caregiver distress. In future research, it is important to study the effects in persons with more complaints and higher distress and to be careful in the selection of outcome variables in relation to the reminiscence functions served by the intervention.

## Introduction

As there is still no treatment for dementia, dementia care mainly focuses on maintaining quality of life and reducing psychosocial problems [1]. The most applied non-pharmacological care consists of behavioral interventions for persons with dementia as well as their caregivers and care environment in order to reduce–and respond to–behavioral changes due to dementia and accompanying neuropsychiatric symptoms (NPS) [2–5]. NPS have a high prevalence amongst persons with dementia [6]. Dementia and the related NPS not only affect the quality of life of persons with dementia, but also lead to a higher level of distress and a lower quality of life of informal caregivers [7–9]. This distress includes physical, emotional and economic aspects [10]. NPS are among the most important reasons for nursing home admittance, as they often make the care at home too burdensome [11]. A systematic review of Olazarán and colleagues shows that NPS can be substantially diminished by behavioral interventions [12]. It is important that such behavioral interventions are person-centered, so that they can meet the needs of persons with dementia and their informal caregivers [13]. This paper describes a randomized controlled trial on such a person-centered behavioral intervention for people with (very) mild dementia: the Online Life Story Book (OLSB).

Reminiscence, which involves the active retrieval of personal memories, is a person-centered behavioral approach [14]. Personal memories are shaped by the autobiographical memory system, which remains intact for a relatively long time despite the progress of the disease [15, 16]. Previous research has shown that reminiscence activities can contribute to the mental health and quality of life of persons with dementia [17–20].

The creation of a life story book (LSB) is a common approach in reminiscence [21]. Important life events, milestones and specific precious personal memories can be included in a LSB. A recent systematic review on LSBs for people with dementia shows an increase in studies in this field and first effects on autobiographical memory, mood, quality of life and relationships [22]. It also gave insight in the diversity of approaches to create a LSB. For example, the LSBs were created mostly in on average six individual sessions in nursing home settings with a range from 3 to 16 sessions. Whilst some studies only focused on the person with dementia, others also examined (in)formal caregivers and found potential effects on the caregiver distress and quality of life. Only three of the most recent studies incorporated a form of technology: one consisted of a movie and the other two of (basic) digital applications with pictures and sounds. This systematic review seems to confirm the previous conclusion of Lazar and colleagues [23] that using technology in reminiscence interventions is promising, but that there is a lack of systematic studies.

The Online Life Story Book (OLSB) is a new reminiscence intervention that allows the user to digitally share memories using multimedia and multisensory cues which might become more important to elicit memories when the disease progresses [23]. Next to the novelty of using technology, our current project is one of the first to conduct an RCT that examines a LSB intervention in the home situation and involves trained volunteers who support creating the digital LSBs. Moreover, effects on the person with dementia as well as their informal caregivers are assessed. Hence, the aim of this study was to investigate the effectiveness of the OLSB on neuropsychiatric symptoms in persons with (very) mild dementia and the distress and quality of life of their primary informal caregivers.

## Methods

### Design

A two-arm randomized controlled trial with individual randomization and three measurements at baseline (T0), 3 months (T1) and 6 months (T2) after baseline was conducted. A detailed description of the study design, intervention and outcome measures is published in a research protocol [24]. This study has been approved by the Twente Medical Ethics Committee under the file number p16-04 (Dutch Trial Register: NTR5939, date of registration: 14 March 2016).

#### Experimental condition: Online Life Story Book

The Online Life Story Book is an e-health application that allows placing personal memories on a dynamic timeline. The timeline is easily marked with historical years and expands as more memories are added. Memories like life events, anecdotes, photos, movies, voice fragments, music, recipes, preferences, and activities can be placed on the timeline. The initial application that was used in this study was developed by Hellomydear. Since this application–unexpectedly–was no longer available during the last couple of months of the study, some participants had to switch to another application. We used Albelli, a commercial application that can be used to create several kinds of photo albums. In Albelli, no timeline is generated, but books were still made in chronological order. Both applications allowed to print the online books. QR-codes made it possible to access the online multisensory memories. The website of Hellomydear is no longer operational.

Trained volunteers supported the persons with dementia and their caregivers (in the following referred to as *dyad*) in making the OLSB. This is more cost-effective for care institutes and easier to organize compared to care provided by professionals like psychologists [25]. Furthermore, an intervention delivered by a volunteer instead of a care professional can be less stigmatizing, as volunteers provide a contact with society rather than with health care professionals [26]. The volunteers visited the dyads approximately five times within a period of 8–10 weeks. The volunteers followed communication guidelines with regard to dementia and reminiscence. They asked dyads about specific milestones and important memories, and nudged them to tell about it as explicit as possible. The volunteers tried to get a variety of memories from different phases of life. Persons with dementia and their family members collected materials that the volunteer digitized when necessary and uploaded in the OLSB.

Thirteen volunteers– 9 women and 4 men–were recruited through local organizations in care and social work. Their age ranged from 28 to 60 years. They had different professional backgrounds in either health care, social work, or technology. The volunteers received four hours training on reminiscence, dementia, conversation techniques, and on how to use the application. The training was led by TE, CU and a senior psychologist. After the switch in application, volunteers were retrained to use the new application and given an updated manual with instructions. During the intervention, volunteers could ask questions and share their experiences via telephone or email or at monthly supervision meetings (led by TE and CU). By excluding persons with a past psychotrauma, having all conversations with both the person with dementia and their informal caregiver so they would feel more safe, and the possibility for the volunteers to consult the researchers and a senior psychologist during the intervention, the potential for distress for the person with dementia was accounted for.

#### Control condition: Wait list with care as usual

The dyads in the control condition received care as usual and were offered to create an OLSB after a period of six months. They were handed out an information letter with possible support and activities for persons with (mild) dementia in the region of Twente. In the Netherlands, usual care for persons with mild dementia consists of care provided by the general practitioner, case management (by the general practice or a nurse practitioner), medication (if indicated) and access to formal care. During this study, no restrictions were placed regarding the care or support dyads requested for.

### Participants

Each person with dementia was accompanied by an informal caregiver; together they formed a dyad.

#### Recruitment and setting

Persons with (very) mild dementia living at home in the region of Twente and being cared for by an informal caregiver were included. The dyads were recruited through local organizations that work with persons with dementia and their informal caregivers (care and social work; general practitioners; memory clinic; informal meetings with peers). Furthermore, articles in local newspapers and door-to-door papers, and a promotional video were used.

#### Inclusion and exclusion criteria

In order to be eligible to participate in this study, a person with dementia had to meet the following criteria: (1) living at home and receiving informal care; (2) having (very) mild dementia (scoring 0.5 or 1 on the Clinical Dementia Rating (CDR) [27]); (3) being mentally capable to provide informed consent (assessed by researcher during intake). A potential participant was excluded when past psychotrauma was present (assessed with the module posttraumatic stress disorder of the Mini International Neuropsychiatric Interview (MINI) [28]).

#### Power analysis

A small effect was expected for the primary outcome at follow-up [19, 20]. The power calculation indicated 74 participants (GPower: f = 0.15; alpha = .05; power = .80; repeated measures ANOVA with 2 groups and 3 measurement points; r = .50 between measurements). Given the vulnerability of the participants and a high mortality rate, a drop-out of 30% was expected [18–20]. Hence, 106 participants needed to be included, 53 per condition.

### Procedure

Most people were approached by informed care professionals who brought them into contact with the researchers. The persons with dementia or informal caregivers who were interested in the project or care professionals who knew people who might be eligible, sent their contact information to the primary investigator (TRE). Then, an information letter about the aim of the project, the eligibility, the process of participation, the benefits and investment of participating, data-management and contact information was sent and an intake was planned. The intake as well as all further meetings for data collection took place at the participants home and were conducted by the researchers. During intake, the project was explained again, questions were being answered and if the participant was still willing to participate, an informed consent was signed (by both the person with dementia and the informal caregiver). This took about 30–40 minutes. Directly after the participant was screened on the inclusion and exclusion criteria, the baseline (T0) measurement was assessed. The total duration of the three assessments was estimated between 90–120 minutes, for both the person with dementia and the informal caregiver.

Eligible dyads were randomized to either the intervention or the wait list control condition. The random allocation sequence was created a priori by a computer-generated randomized number list with stratification on gender of the person with dementia (randomizer.org). When randomized to the experimental group, a volunteer was assigned to the dyads. When allocated to the wait list control group, a volunteer was assigned six months after intake after all study measurements had ended. The first inclusion measurement was assessed in June 2016 and the last measurement took place in December 2017. The trial ended because the project lasted from February 2016-February 2018, so all data needed to be gathered before February 2018.

From both an ethical and practical point of view, it was impossible to keep the dyads blinded to the allocation. As stated above the persons in the wait list control condition received care as usual and were handed out an information letter with possible support and activities for persons with (mild) dementia in the region.

### Measures

#### Characteristics of participants

The sociodemographic and health characteristics of the participants were assessed with parts of The Older Persons and Informal Caregivers Survey Minimum DataSet (TOPICS-MDS [29]). The following socio-demographics of both the persons with dementia and their informal caregivers were assessed: sex, age, education, and marital status. Health for persons with dementia was measured with a questionnaire on the presence or absence of 17 common diseases; the Katz-15-ADL [30] that asks for the need for support for activities of daily living; a question of the RAND-36 [31] that measures interference of physical and emotional problems with social activities; a single question on subjective health; five questions of the RAND-36 [31] that measure psychological well-being; one question of the RAND-36 [31] about quality of life in general; and a variant of Cantril’s Self Anchoring Ladder [32] in which persons were asked to rate their life satisfaction on a scale from 0–10. Health for informal caregivers was measured only with a single question on subjective health.

#### Primary outcome

*Neuropsychiatric symptoms (NPS)*. To assess the effect of the intervention on the primary outcome, neuropsychiatric symptoms, the Neuropsychiatric Inventory (NPI) was assessed at all three time points in all participants [33, 34]. The NPI is a reliable and valid measure that assesses the frequency, severity and distress of twelve neuropsychiatric symptoms: delusions, hallucinations, agitation/aggression, depression/dysphoria, anxiety, elation/euphoria, apathy/indifference, disinhibition, irritability/lability, motor disturbance, nighttime behaviors, and appetite/eating. The frequency (F) is provided on a scale from 0 = never to 4 = daily, the severity (S) on a scale from 0 = not to 3 = severe. The score for each of the twelve symptoms is computed as the frequency multiplied by the severity, resulting in a score ranging from 0 to 12. An FxS score of 4 or higher is considered as clinically relevant. The scores on the individual symptoms were also summed towards scores on four symptom clusters: hyperactivity, psychosis, affective symptoms, and apathy [35]. Last, the twelve FxS scores were added to a total score (0–144 [35]). For the NPI symptom clusters and the total score there is no clinical cut-off score, because of the many disparate behaviors [33].

#### Secondary outcomes

*Caregiver Distress*. *General caregiver distress* was measured with a scale on the perceived distress in informal care (EDIZ [36]). The informal caregiver rated the subjective distress on nine items with a five-point scale. The answers were dichotomized per item (no! and no = 0; more or less, yes and yes! = 1) and then added up to a score between 0 and 9.

*Caregiver distress due to neuropsychiatric symptoms* was measured with the distress scales of the NPI [33, 34]. The caregiver rated the distress for each of the twelve neuropsychiatric symptoms on a scale from 0 = none to 5 = severe. These twelve distress scores were summarized to a total score, ranging from 0–60. This sum score provides an indication of the emotional distress caused by all neuropsychiatric symptoms.

The questions on Distress and Time investment of the TOPICS-MDS [29] were used to assess caregiver distress. The *self-rated distress* was measured with a single question where caregivers rated the distress of care to the person with dementia on a scale from 0 to 100. The *time investment* is the total hours per week that the informal caregiver spent to assist the person with dementia with household tasks, personal care, and moving outside the house.

*Quality of life of the caregiver*. The quality of life was assessed with parts of the TOPICS-MDS [29]. The *care-related quality of life* of the caregiver was measured with the CarerQol [37], which consists of seven questions that are rated on a three-point scale (1 = none to 3 = many) as well as a visual analogue scale on happiness ranging from 0 to 10. The *general quality of life* of the caregiver was assessed with a question of the RAND-36 [31] and *life satisfaction* was measured with a variant of Cantril’s Self Anchoring Ladder [32], in which persons were asked to rate their life on a scale from 1–10 (item: ‘*What grade do you give your life at the moment*?*’*).

### Statistical analyses

All statistical analyses were performed using SPSS 25.0 (IBM SPSS Statistics). All tests were two-tailed using a 95% confidence interval. First, frequency distributions were made of all sociodemographic and health characteristics as well as the baseline assessments of neuropsychiatric symptoms, caregiver distress, and caregiver quality of life. Second, in order to assess the success of the randomization, the baseline characteristics between the two conditions were analyzed with χ^2^ tests and t-tests. Third, to assess selective drop-out, χ^2^ tests and t-tests were used to compare persons who did or did not complete the whole study.

To analyze the primary and secondary outcomes of the intervention, we used a mixed model analysis that allows to take all existing information into account, even in spite of the fact that some participants dropped out. A random within-subjects effect was modeled as a repeated measure with correlated residuals. We specified two fixed factors: condition (intervention versus control) and time (baseline, 3 months, 6 months). Because of the differences between the two applications we also conducted analyses with three condition levels (Hellomydear, Albelli, and control). We tested several covariance types for the within-subjects factor and used the model fit (Akaike Information Criterion and Baseysian Information Criterion) to find the best fitting type. This was either the unstructured or the heterogeneous first-order autoregressive type. We report the expected marginal means and used those at six months follow-up to compute the Cohen’s *d* between the conditions (below .33 is interpreted as small; between .33 and .55 is moderate; above .55 is large [38]). We also carried out repeated measures analyses (General Linear Model) with completers only, with ‘time’ as within subject factor, ‘condition’ as between subject factor as well as the interaction between the two. As these analyses showed the same significant findings, we decided to only report the results of the mixed model analyses.

## Results

### Participant flow

**Fig 1:**
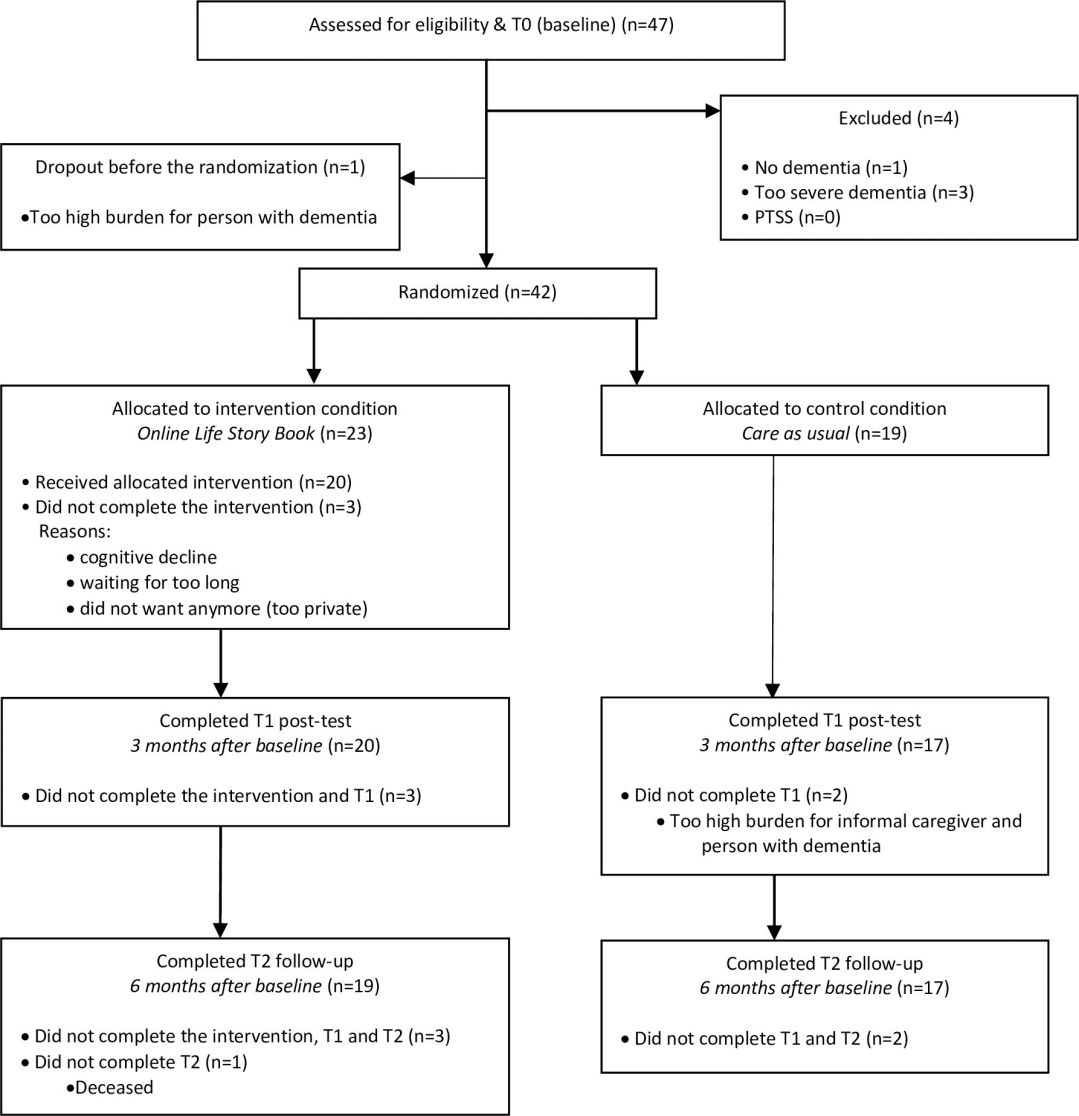
Participant flow.

Fig 1 presents the details on the participant flow. Of the 47 participants assessed for eligibility, 42 were included and randomized. One person was excluded because of no dementia (CDR score of 0) whereas three were excluded because of too severe dementia (CDR score above 1). Before randomization, one informal caregiver reported a too high burden for the person with dementia to take part in the study.

The actual drop-out of participants during the entire project was 14.3 percent. There was no significant difference between the intervention and the control condition (χ^2^ (1) = 0.4; p = .527). Of the 23 participants randomized to the intervention condition, four (17.4%) did not complete the intervention and the study, because of several reasons: one had a fast cognitive decline, so it would be too much of a burden to create the online life story book; one had to wait for too long to be assigned to a volunteer; one changed his mind because he found it too private to share memories; and one person passed away before the follow-up measurement. Of the 19 participants allocated to the control condition, two (10.5%) did not complete the measurements at three and six months, because of a too high burden for the informal caregiver and the participant.

### Participant characteristics

#### Persons with dementia

Table 1 presents the baseline characteristics of the persons with dementia. Of the 42 participants, 23 were female and 19 were male. They had a mean age of 80.6 years (SD = 9.4), ranging from 49 to 95 years. Fifty percent had finished primary or lower vocational education, 30% secondary or middle vocational education, and 20% higher education. Participants were married (69%) or widowed (31%). They had very mild (26%) or mild dementia (74%) according to the clinical dementia rating scale. They indicated on average to have 4.1 (SD = 1.8) out of 17 diseases and to need help on 6.9 (SD = 4.0) out of 15 domains of functioning. Their social activities were sometimes impeded because of physical or emotional problems (mean = 3.2; SD = 1.2; scale 1–5 with 1 being continuously impeded and 5 being never). Their subjective health was rated as moderate to good (mean = 2.5; SD = 0.8; scale 1–5); their psychological well-being score was on average 23.6 (SD = 3.3; scale 5–30); their life satisfaction was 7.5 (SD = 1.0; scale 0–10); and their self-rated quality of life was good (mean = 3.0; SD = 0.9; scale 1–5). With regard to the primary outcome measure, they scored on average 9.2 (SD = 10.1; scale 0–144) on the frequency by severity scale of the Neuropsychiatric Inventory. For hyperactivity, the average score was 0.6 (SD = 1.0), for psychosis 0.6 (SD = 0.8), for affective symptoms 1.1 (SD = 1.3) and for apathy 1.1 (SD = 1.7). Few participants scored above the cut-off for clinically relevant complaints (0 on delusions, 0 on hallucinations, 4 on agitation/aggression, 2 on depression/dysphoria, 3 on anxiety, 1 on elation/euphoria, 4 on apathy/indifference, 2 on disinhibition, 4 on irritability/lability, 4 on motor disturbance, 5 on nighttime behaviors, and 9 on appetite/eating). However, 48% of the participants with dementia had clinically relevant symptoms on at least one of the twelve symptoms.

**Table 1:** Demographical data and personal information of persons with dementia at baseline.

Variable	All	OLSB	Control	t/ χ (df)
	(N = 42)	(N = 23)	(N = 19)	
**Age, mean in years (SD)**	80 (9.4)	79.5 (8.1)	81.2 (11.2)	0.6 (40)^a^
**Female N (%)**	23 (55.8%)	16 (69.6%)	7 (30.4%)	4.5 (1)*
**Educational level N**	40	22	18	
*Primary and lower vocational education*	20 (50.0%)	13 (59.1%)	7 (38.9%)	1.9 (2)^b^
*Secondary and middle vocational education*	12 (30.0%)	6 (27.3%)	6 (33.3%)	1.9 (2)^b^
*Higher education*	8 (20.0%)	3 (13.6%)	5 (27.8%)	1.9 (2)^b^
**Marital status**				
*Married*	29 (69.8%)	14 (60.9%)	15 (79%)	1.6 (1)^b^
*Widowed*	13 (30.2%)	9 (39.1%)	4 (21%)	1.6 (1)^b^
**Cultural background**				
*Born in the Netherlands*	39 (93%)	21 (91.3%)	18 (94.7%)	0.2 (1)^b^
*Born abroad*	3 (7%)	2 (8.7%)	1 (5.3%)	0.2 (1)^b^

Note.

^a^ No significant differences between intervention and control condition (t-test with p> 0.05).

^b^ No significant differences between intervention and control condition (χ2-test with p> 0.05).

* Significant differences at baseline between both groups (p< 0.05).

#### Informal caregivers

Table 2 presents the baseline characteristics of the informal caregivers. Of the 42 informal caregivers, 31 were female and 11 were male. They had a mean age of 62.8 years (SD = 13) ranging from 38 to 88 years. Twenty of the informal caregivers were a spouse who lived together with the person with dementia, whereas twenty-two were family members, such as a child, niece or nephew, who did not live together with the person with dementia. Their subjective health was good to very good (mean = 3.4; SD = 1.0; scale from 1–5). With regard to the measures of caregiver distress at baseline, their general distress was on average 4.0 (SD = 2.5; scale from 0–9), the average distress due to neuropsychiatric symptoms was 5.9 (SD = 6.1, scale 0–60), and the average self-reported distress was 39.7 (SD = 26.9; scale 0–100). Caregivers spent on average 11.8 hours per week (SD = 15.2) caring for the person with dementia. With regard to caregiver quality of life, they rated their quality of life at baseline as good to very good (mean = 3.7; SD = 0.9; scale from 1–5) and their average life satisfaction as 7.5 (SD = 1.2 on a scale from 0–10).

**Table 2:** Demographical data of informal caregiver.

	All	OLSB	Control	t/ χ (df)
	(N = 42)	(N = 23)	(N = 19)	
**Age, mean in years (SD)**	63 (13)	63 (13)	63 (14)	0.1 (36)^a^
**Female N (%)**	31 (73.8)	15 (65.2)	16 (84.2)	1.9 (1)^b^
**Spouse and living together**	20 (47.6)	12 (52.2)	8 (42.1)	0.8 (2)^b^
**Health, 1–5 (SD)**	2.5 (1.0)	2.6 (1.0)	2.5 (1.0)	-0.1 (40)^a^

Note.

^a^ No significant differences between intervention and control condition (t-test with p> 0.05).

^b^ No significant differences between intervention and control condition (χ2-test with p> 0.05).

#### Randomization check

Except for the gender of the person with dementia, there were no significant differences between the intervention and control condition on any of the demographic and health variables of the persons with dementia and their caregivers (all χ^2^ tests and t-tests had p>.05). There were also no significant differences in the total baseline scores and the four domain baseline scores of neuropsychiatric symptoms between the intervention and the control condition (t-tests with p>.05). Last, there were no significant differences in any of the measures of distress or quality of life of the caregivers at baseline (t-tests with p>.05).

#### Drop-out analyses

There were no significant differences between the persons who did (n = 36) or did not (n = 6) complete all study measurements on any of the demographic and health variables of the persons with dementia and their caregivers (χ^2^ tests and t-tests with p>.05). There were also no significant differences in neuropsychiatric symptoms at baseline, nor in caregiver distress or caregiver quality of life at baseline (t-tests with p>.05). In sum, there was no selective drop-out.

### Primary outcome

Table 3 presents the estimated marginal means of the mixed model analyses for the primary outcome neuropsychiatric symptoms. There were no significant effects of time, condition, or their interaction. The significant difference in gender of the person with dementia between the two conditions at baseline did not affect these outcomes: no significant effects of time, condition, or their interaction were found. As not all participants in the intervention condition received the same kind of online life story book, we also carried out a mixed model analysis on three conditions (Hellomydear, Albelli, and control). Again, there were no significant effects of time, condition, or their interaction. When analyzing the different clusters of neuropsychiatric symptoms (Hyperactivity, Psychosis, Affective Symptoms and Apathy) separately, no significant differences were found for condition, time, or their interaction. All effect sizes were small at six months follow-up with the exception of the effect size for psychosis which was moderate.

**Table 3:** Results on primary outcome neuropsychiatric symptoms (estimated marginal means).

		Baseline	3 Months	6 Months	6 Months	Condition	Time	Interaction
		Mean (SE)	Mean (SE)	Mean (SE)	Cohen’s d	F(1)	F(2)	F(2)
Neuropsychiatric	Intervention	8.7 (2.4)	9.8 (2.5)	12.2 (2.6)	-0.03	0.3	1.9	1.1
Symptoms	Control	9.8 (2.7)	13.6 (2.7)	12.5 (2.8)				
Neuropsychiatric	Hellomydear	8.8 (3.4)	7.3 (4.0)	13.4 (4.3)	0.09	0.1	1.6	1.8
Symptoms	Albelli	8.6 (2.8)	11.9 (3.4)	10.9 (3.8)	-0.07			
	Control	9.8 (2.4)	13.6 (2.8)	12.5 (3.1)				
Hyperactivity	Intervention	3.4 (1.1)	4.4 (1.3)	4.3 (1.7)	-0.04	0.1	1.1	1.2
	Control	3.0 (1.0)	3.3 (1.2)	4.0 (1.6)				
Psychosis	Intervention	1.2 (0.5)	1.9 (0.7)	0.9 (0.4)	0.40	0.5	2.2	0.8
	Control	2.1 (0.5)	1.7 (0.6)	1.5 (0.4)				
Affective	Intervention	4.7 (1.2)	6.6 (1.4)	5.8 (1.3)	0.07	0.2	0.9	1.4
Symptoms	Control	4.4 (1.1)	4.6 (1.3)	6.1 (1.2)				
Apathy	Intervention	5.1 (1.5)	6.4 (1.6)	6.1 (6.1)	0.18	0.1	2.0	1.8
	Control	3.5 (1.4)	5.1 (1.4)	7.0 (1.6)				

No significant differences.

### Secondary outcomes

Table 4 presents the estimated marginal means of the mixed model analyses for the secondary outcomes caregiver distress and caregiver quality of life. With regard to the secondary outcome measures, only the interaction effect for self-rated distress is significant (F(2) = 3.2; p = .045). At three months follow-up caregivers in the intervention condition report somewhat less distress than caregivers in the control condition, but at six months slightly more. The effect sizes at six months follow-up are small with the exception of moderate effect sizes for time investment and general quality of life.

**Table 4:** Results on secondary outcomes caregiver distress and caregiver quality of life (estimated marginal means).

		Baseline	3 Months	6 Months	6 Months	Condition	Time	Interaction
		Mean (SE)	Mean (SE)	Mean (SE)	Cohen’s d	F(1)	F(2)	F(2)
General distress	Intervention	3.8 (0.5)	3.8 (0.6)	3.9 (0.6)	0.20	0.1	1.1	1.6
	Control	4.1 (0.6)	3.3 (0.7)	3.5 (0.7)				
Distress neuro-	Intervention	5.5 (1.3)	5.4 (1.5)	6.6 (1.8)	-0.18	0.4	1.0	1.4
psychiatric symptoms	Control	6.4 (1.4)	7.3 (1.6)	7.7 (1.9)				
Self-rated distress	Intervention	38.9 (5.8)	31.8 (5.9)	44.7 (6.1)	0.18	0.1	2.8	3.2*
	Control	40.7 (6.3)	41.4 (6.5)	41.1 (6.5)				
Time investment	Intervention	12.3 (3.2)	13.9 (3.7)	16.7 (4.8)	0.37	0.2	1.0	1.3
	Control	11.3 (3.5)	13.2 (4.1)	10.7 (5.3)				
Care-related quality	Intervention	17.5 (0.5)	17.5 (0.6)	17.2 (0.6)	-0.20	0.0	0.9	1.0
of life	Control	17.1 (0.6)	17.7 (0.7)	17.6 (0.6)				
General quality of life	Intervention	2.5 (0.2)	2.5 (0.2)	2.5 (0.2)	-0.43	1.8	0.0	0.0
	Control	2.8 (0.2)	2.9 (0.2)	2.8 (0.2)				
Life satisfaction	Intervention	7.7 (0.2)	7.8 (0.2)	7.5 (0.3)	-0.02	0.3	0.8	0.3
	Control	7.4 (0.3)	7.6 (0.3)	7.5 (0.3)				

* p < .05.

## Discussion

This study is one of the first to conduct an RCT to examine the effects of an Online Life Story Book created in the home setting by volunteers for people with (very) mild dementia and their caregivers. Contrary to expectations the results show no significant differences between the experimental condition and the wait list control condition with self-rated distress of informal caregivers being the only exception.

There could be several reasons to explain these results. To start with, persons with dementia reached in our study appeared to have few neuropsychiatric symptoms whereas caregivers did not perceive much distress and reported a relatively high quality of life compared to other studies with the same target group [39–42]. This may have caused floor and ceiling effects so there was almost no room for improvement. The preventive effect of the OLSB could have become visible if an extra follow-up at 12 months was assessed, as normally NPS are expected to develop over the course of time. Related to this, our open recruitment may have led to reaching a specific group of persons with (very) mild dementia and their informal caregivers: only people that were initially motivated and felt capable enough to participate did sign up. The setting might have been important too: a meta-analysis on reminiscence therapy found greater improvement on depressive symptoms for institutionalized people with dementia than community-dwelling people with dementia [17] and according to a recent Cochrane systematic review on reminiscence therapy for dementia the impact on quality of life appeared most promising in care home settings [43]. So it could be that it is harder to measure change in people with dementia living at home than in relatively more standardized and homogenous care home settings. Hence, the question remains how the intervention would have worked for people with more severe complaints, or who did not feel the competence to participate in such a project.

Next, outcomes might not have been significant because less persons participated than initially anticipated, even though the Cohens d corresponded to the small effect sizes that were assumed in the power analysis. Finding participants that suffered from i) mild dementia, ii) were living at home iii) had an informal caregiver that wanted to actively contribute iv) felt the space and competence and v) wanted to talk about personal matters appeared to be a real challenge. Despite the smaller number of participants, the drop-out during the intervention was substantial lower than expected, resulting in 85.7% completers versus the anticipated 70%.

The persons that withdrew, did so mostly because of reasons that were not related to the intervention itself. The switch in application could have biased the results as the time periods between baseline and T1 were extended for those dyads. However, only one person refrained because of waiting too long before the new application could be used. Most dyads did not mind the change in application to create the OLSB and controlling for type of application did not show any difference in significant levels over time. Having said that, the change was time consuming, people had to wait somewhat longer to start, or people who were already in the process of making the OLSB had to wait and start over with the new application.

Another reason for the unexpected outcome of no effect, could also be due to the kind of outcome variables that have been used. As can be seen in the review of Elfrink and colleagues, existing research on LSBs for persons with dementia focused on different types variables such as cognition, mood (depression), quality of life and communication/quality of caregiving relationship [22]. Many of those variables have also been evaluated on their effectiveness in the Cochrane systematic review on reminiscence and dementia, but due to the diversity of study designs (group vs individual approach; care home vs home setting) and outcome measures a proper comparison was hard to make. That may have caused the small and inconsistent effects that the researchers found in their review [43]. Most of these outcomes have not been covered in our study, since we were especially interested in NPS and caregiver distress which can be seen as more distal outcomes, as it takes more time to see an effect in those variables. Nevertheless, we strongly believe that incorporating outcomes on caregiver distress and quality of life of the caregiver is a strength in our research since dementia not only affects the person with dementia but the family or system as a whole. In line with this, research has shown that reminiscence can have different functions (i.e. identity construction, problem solving, and death preparation, bitterness revival, boredom reduction, and intimacy maintenance, conversation and teaching or informing) and that is important to match these functions with the aim of the reminiscence intervention and consequently the outcome measures [14]. It could be that the OLSB predominantly serves the social functions and less explicitly the more therapeutic functions. To summarize, the question remains which kind of outcome variables should be included in reminiscence interventions for persons with dementia, how to assess them and what time-frame of measurement fits best for which variable.

Next to the effectiveness of this intervention it is also important to take a look into the usefulness and feasibility. For instance, it can be a real advantage that persons with dementia low on burden of disease–like in our study–are able to actively contribute to their own life story book, which may result in a more personal story of their own. The fact that there was a very low drop-out rate and that those dyads that withdrew did so mainly because of reasons that were not related to the intervention itself, would suggest that the dyads accepted the intervention.

Including experienced changes by those involved could help to get a better understanding of the potential benefits of reminiscence interventions. To gain better insight in the perceived efficacy and implementation from the perception of different stakeholders (participants, informal caregivers, volunteers and care professionals) a process evaluation was conducted parallel to this RCT. These complementary results will be described in a following paper (in preparation).

## Conclusion

Despite the absence of significant effects on the use of an Online Life Story Book for people with (very) mild dementia and their informal caregivers, this study contributes to the research on LSBs and does provide valuable implications. It shows that in future research, it is important to study the effects in persons with more complaints and higher burden and to be careful in the selection of outcome variables in relation to the reminiscence functions served by the intervention.

## Supporting information

S1 Checklist CONSORT 2010 checklist of information to include when reporting a randomised trial*. (DOC)

## List of abbreviations

CDR: Clinical Dementia Rating
EDIZ: Ervaren Druk door Informele Zorg
LSB: Life story book
MINI: Mini International Neuropsychiatric Interview
NPI: Neuropsychiatric Inventory
NPS: Neuropsychiatric symptoms
OLSB: Online Life Story Book
QOL: Quality of life
RCT: Randomized controlled trial
SD: Standard deviation
TOPICS-MDS: The Older Persons and Informal Caregivers Survey Minimum DataSet
